# Improved Time Resolved KPI and Strain Characterization of Multiple Hosts in Shake Flasks Using Advanced Online Analytics and Data Science

**DOI:** 10.3390/bioengineering9080339

**Published:** 2022-07-25

**Authors:** Rüdiger W. Maschke, Barbara Pretzner, Gernot T. John, Christoph Herwig, Dieter Eibl

**Affiliations:** 1 Institute of Chemistry and Biotechnology, School of Life Sciences and Facility Management, ZHAW Zurich University of Applied Sciences, Grüentalstrasse 14, 8820 Wädenswil, Switzerland; dieter.eibl@zhaw.ch; 2 Körber Pharma Austria GmbH, Mariahilfer Straße 88A/1/9, 1070 Vienna, Austria; christoph.herwig@koerber.com; 3 Research Area Biochemical Engineering, Vienna University of Technology, Gumpendorfer Strasse 1a, 1060 Vienna, Austria; 4 PreSens Precision Sensing GmbH, Am BioPark 11, 93053 Regensburg, Germany; g.john@presens.de; 5 Competence Center CHASE GmbH, Altenbergerstraße 69, 4040 Linz, Austria

**Keywords:** shake flask, key performance indicator, strain characterization, online-analytics, growth rate estimation, specific oxygen consumption, microbial cultivation, plant suspension cultures, mammalian cell cultures, optrodes

## Abstract

Shake flasks remain one of the most widely used cultivation systems in biotechnology, especially for process development (cell line and parameter screening). This can be justified by their ease of use as well as their low investment and running costs. A disadvantage, however, is that cultivations in shake flasks are black box processes with reduced possibilities for recording online data, resulting in a lack of control and time-consuming, manual data analysis. Although different measurement methods have been developed for shake flasks, they lack comparability, especially when changing production organisms. In this study, the use of online backscattered light, dissolved oxygen, and pH data for characterization of animal, plant, and microbial cell culture processes in shake flasks are evaluated and compared. The application of these different online measurement techniques allows key performance indicators (KPIs) to be determined based on online data. This paper evaluates a novel data science workflow to automatically determine KPIs using online data from early development stages without human bias. This enables standardized and cost-effective process-oriented cell line characterization of shake flask cultivations to be performed in accordance with the process analytical technology (PAT) initiative. The comparison showed very good agreement between KPIs determined using offline data, manual techniques, and automatic calculations based on multiple signals of varying strengths with respect to the selected measurement signal.

## 1. Introduction

The creation of a biopharmaceutical product from the very first development steps to market maturity costs several millions, if not billions, of euros [1,2]. To minimize the risk to patients and subsequent product failure, it is essential to thoroughly understand the product as well as the production process and its impact on product quality. For this reason, regulatory bodies such as the U.S. Food and Drug Administration (FDA) or the European Medicines Agency (EMA) require proof that the product and the process have been sufficiently investigated by the manufacturers. In the last two decades, the concept of quality by design (QbD), which has also been included in the International Conference on Harmonization (ICH) guidelines, has proven to be helpful and has become accepted globally [3,4,5,6]. According to ICH, the QbD approach includes the definition of a quality target product profile (QTPP), from which critical quality attributes (CQAs) are derived [7]. Based on prior knowledge and risk assessments, potentially critical process parameters (PCPP) are identified, whose effects on the CQAs are studied in more detail using statistical analyses, such as one factor at a time (OFAT) or design of experiments (DoE) methods [8,9,10]. Then, based on the results of these statistical analyses, a control strategy can be set for the process using, for example, design spaces or proven acceptable ranges (PAR) [11,12]. More and more manufacturers are applying aspects of QbD into their process development. However, this occurs relatively late in the engineering phase and focuses more on the downstream processing (DSP) [13,14]. The early-stage development of upstream processing (USP) is more often based on experience or textbook knowledge instead of sound knowledge management and QbD [15]. USP in particular, with all its possible parameters, such as strain/cell line, production medium, and cultivation conditions, greatly influences the quality of the product [16,17]. It is, therefore, possible to determine PCPPs associated with the cell–medium complex in initial small-scale cultivations [18]. Process-oriented strain/cell line and media characterization at this scale can be used to compare different approaches and subsequently develop a scale-up process based on QbD. The most important comparison parameters can be defined as key performance indicators (KPIs). Often used KPIs are the maximum specific growth rate, µ_max_, the specific oxygen uptake rate, q_O2_, and yields (biomass related to substrate Y_X/S_ or product related to biomass Y_P/X_) for a process-oriented strain/media characterization.

Strain/cell line and medium characterization are still most frequently performed in shake flasks, justified by their low costs, ease of use, and ability to be run in parallel [19,20,21]. This removes the complex preparation requirements of traditional bioreactors as well as the high costs of using stirred single-use systems with control units [22]. Despite these evident advantages, automated mini-bioreactors [23,24] and well plates [25,26,27] are more often used than shake flasks for this purpose because of one major weakness of this cultivation system: the lack of standardized online measurement and control, making the application of QbD in early-stage development more difficult for shake flasks. This deficiency has two significant consequences. 

Firstly, it is not possible to adapt cultivation process parameters to changing conditions within the shake flask. For example, the pH in mammalian cell cultivations can be kept within a physiologically acceptable range for many applications via a carbonate buffer system and the creation of a CO_2_ atmosphere in the incubator. An elevated pCO_2_ concentration can result in reduced cultivation success due, for example, to the absence or delayed occurrence of the lactate shift [28]. Moreover, for fast-growing cultures such as *E. coli* with elevated substrate (e.g., glucose) concentrations, overflow metabolism can be detected [29], leading to increased and inhibiting levels of acetate [30,31] which may exceed the buffer capacity, resulting in decreasing pH-values [32]. 

This example immediately illustrates the second issue associated with shake flask cultivations: the lack of ability to perform online measurements, or more precisely standardized and comparable application of measurement techniques. Obviously, online data can be recorded, and this is usually conducted for the entire shaker using manufacturer specific software (e.g., eve for Infors HT or Insight for Kühner AG shakers). In an effort to record not only data in the incubator (such as shaking rate or temperature), supplementary measuring systems have also been developed. Several approaches exist to describe the properties in shake flasks during cultivation: the RAMOS [33,34,35,36,37], Kuhner TOM [38] and BCPreFerm [39] systems using off-gas measurement; the cell growth quantifier using backscattered light (BSL) [40,41]; the SENBIT system using a multiparameter probe [42]; the Shake Flask Reader SFR using shake flasks equipped with pH and DO sensor spots; the CITSens Memo performing glucose and lactate measurement [43]; and the Shake Flask Reader SFR vario, combining DO, pH, and CO_2_ sensor spots with backscattered light measurements [44,45].

All these developments simplify or eliminate manual sampling in shake flasks, thus enabling online monitoring. However, most data analysis, such as KPI calculations, are still performed manually. To facilitate and standardize data evaluation of shake flask experiments and thus also improve the QbD approach, algorithm assisted evaluation techniques can be used [46]. Since most machine learning or artificial intelligence algorithms require a large amount of data to make robust predictions, these algorithms tend not to be suitable as there is rarely enough training data in early-stage development [47].

This paper presents a novel workflow which uses non-invasive online measurements in shake flask experiments and enhanced data science to deliver automated and standardized real time calculation of KPIs for early-stage development experiments. The goal is to standardize media and strain characterization and make it more efficient, thus enabling better comparison between results. This approach consists of the following four steps:

1. Use a recipe database as a basis for knowledge management;
2. Automate and standardize detection of the exponential growth phase within shake flask experiments with enhanced data science;
3. Automate determination of KPIs based on the detected exponential growth phase and data from the recipe database;
4. Store KPIs in the database to simplify and enable comparison with other recipes.

The results were verified in two stages. First, the obtained online KPIs were calculated manually and, where possible, compared with the corresponding offline or literature data. Subsequently, the manually evaluated data were compared with that of the workflow. The variations in application, be it different filling heights, use of baffles, variation in medium, cell size, growth rate, oxygen demand and shaking speed were illustrated using representatives of microbial (*E. coli* and *S. cerevisiae*), plant (*Vitis vinifera*), mammalian (CHO, HEK) and insect cell cultures (High Five). 

## 2. Results and Discussion

### 2.1. Workflow

**Figure 1:**
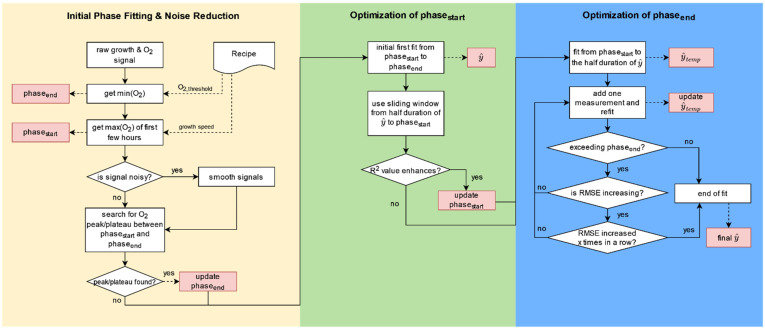
Illustration of the workflow, consisting of three parts, to automatically determine the exponential growth phase in a shake flask cultivation. The first part “Initial Phase Fitting & Noise Reduction” of the workflow, shown in yellow, covers noise detection, signal smoothing if necessary, and the initial setting of the start (phase_start_) and end (phase_end_) of the exponential growth phase as described in Section 2.1.1. The second part “Optimization of phase_start_” of the algorithm, depicted in green and described in Section 2.1.2, optimizes the setting of phase_start_ to improve the exponential fit, thus the predicted value of the exponential growth curve ($\hat{y}$). The last and third part “Optimization of phase_end_” of the workflow shown in blue optimizes the setting of phase_end_ to further improve the exponential fit and produces the final output ($\hat{y}$), as described in Section 2.1.3. The individual steps of the algorithm are marked with white rectangles, outputs are highlighted as red rectangles.

The goal of this workflow, as shown in Figure 1, was to automatically identify the best fit for the exponential growth phase in the OUR or BSL signal. For the exponential fit to best match the observed exponential phase of the selected growth signal (BSL or OUR), it was important that the start, phase_start_, and end, phase_end_, of the exponential growth were set as accurately as possible. Unfortunately, signals are often subject to interference caused by high sensor noise, user errors or poorly chosen settings. The conditions of the shake flasks used, such as baffles or scratches on the flask surface, also contribute significantly to the signal to noise ratio, resulting in the start and end of the phase not being easy to identify. Therefore, the developed algorithm must detect and be robust against such disturbances. To enhance the robustness of the algorithm, a recipe was used, particularly in the initial phase fitting, which advised the algorithm of the approximate time interval when the exponential growth phase wa expected. The recipe contained rudimentary meta information, as shown in Table 1. Except for the two input values in the recipe, this workflow operated automatically without any user input or output.

The different recipes of the tested organisms were only differentiated according to whether the culture had a long or a short cultivation period. Not only was the growth signal itself important for detecting the exponential growth phase, but the information from the oxygen signal was also used, as its characteristics are useful for interpreting growth behavior.

The created workflow consists of three parts. The initial phase fitting and noise reduction, optimization of phase_start_, and optimization phase_end_ are explained in the following sub chapters. 

#### 2.1.1. Initial Phase Fitting and Noise Reduction

First, the raw signal of the OUR or biomass and oxygen are read in. In the second step, the minimum oxygen value is determined, since this characteristic can be the first indication of the end of the exponential growth curve, phase_end_. During cultivation, an oxygen signal usually has several minima, however, in this case only the minima between the beginning of the experiment and the time when the O_2_ signal reaches a certain threshold (O_2, threshold_), when exponential growth is practically no longer possible, is of interest. The O_2, threshold_ can differ from organism to organism, therefore, this information is stored in the recipe. The identified minima of the oxygen signal between the start of the experiment and O_2, threshold_ is then stored as the initial phase_end_.

The maximum value of the oxygen signal can be an indication of the start of the exponential growth curve (phase_start_) and is, therefore, identified in the next step. At the beginning of the cultivation in particular, oxygen may increase sharply in the experimental set-up (e.g., the time needed for temperature and oxygen equilibration after inoculation where the flask stands unmoved at room temperature), leading to a misidentification of the maximum oxygen value. To enhance robustness of this workflow, the search for the maximum value is restricted to a time window relating to an assumed growth speed. A distinction was drawn between slow (*Vitis vinifera*), medium (HEK, CHO) and fast-growing organisms (*E. coli, S. cerevisiae*), as listed in Table 1.

The next step is to check for peaks or plateaus in the timeframe of phase_start_ and O_2,min_ in the growth signal, as these peaks can be another good indicator for a metabolic change and, therefore, for the end of the exponential growth (phase_end_). However, this step is particularly prone to errors if the signal is too noisy. Therefore, a check is performed in advance to establish whether the signal has a high signal-to-noise ratio. If this is the case, the growth signal was smoothed with a Savitzky–Golay filter provided by the SciPy Python package [48,49].

If a peak or a plateau is found in the defined time frame, this characteristic is set as phase_end_. If neither of these two features can be identified, O_2, min_ is stored as phase_end_.

#### 2.1.2. Optimization of Phase Start

At this point, the duration between phase_start_ and phase_end_ is assumed to be the maximum possible exponential growth phase. However, an exponential fit in this initial time period may lead to a poor-quality fit as a result of a low coefficient of determination (R^2^) or a high root mean square error (RMSE). The value for R^2^ can range between 0 and 1 and explains how well the model predicts the observed data in terms of proportion, whereas a value close to 1 is favorable. To increase the quality, and thus also the accuracy of the fit, the setting of phase_start_ and phase_end_ are optimized, as shown in the next parts of the workflow. 

First, an initial fit between phase_start_ and phase_end_ is established, leading to $\hat{y}$. Using a sliding window, a loop operated from half of $\hat{y}$ to phase_start_. In each iteration, the R^2^ value of $\hat{y}$ and the observed growth data within the sliding window are calculated. If the value of R^2^ worsened substantially, the loop is aborted and phase_start_ is moved forward to the time point before the difference between $\hat{y}$ and the signal became too large.

#### 2.1.3. Optimization of Phase End

After phase_start_ was optimized, the setting of phase_end_ can be improved. The first step is to create an exponential, temporary fit between the updated phase_start_ and half of $\hat{y}$, resulting in ${\hat{y}}_{temp}$. In a loop, one after the other, another measuring point is added to ${\hat{y}}_{temp}$ and refitted. With each new fit the RMSE is returned, providing information on how far, on average, the predicted values are from the observed data, whereas a low RMSE was favorable. If the RMSE increased x times in a row, the loop is aborted, since the exponential growth phase is assumed to be over. This query ensures that a local worsening of the fit, caused by noise, does not result in the optimization being aborted too early. The time point before the RMSE becomes deteriorated is set as new phase_end_. If there is no worsening of the RMSE, the phase_end_ remaines unchanged. Lastly, an exponential fit is once again created between the updated phase_start_ and phase_end_, which leads to the final $\hat{y}$.

### 2.2. Cultivation Results

To perform process-oriented strain characterization, as many KPIs as possible should be obtained from online data with minimal manual sampling effort. To allow a uniform evaluation despite the strong differences in the cultivation of plant and animal cell cultures and microbial fermentations, we decided on the following four relevant parameters for strain characterization:

- maximum specific growth rate µ_max_;
- cell-specific oxygen consumption rate q_O2_;
- biomass and product yield Y_X/S_ and Y_P/S_;
- maximum achieved biomass concentration C_X,max_.

However, to determine these parameters, some factors need to be known, for example, the initial biomass C_X,0_ and nutrient C_S,0_ concentrations, as well as the maximum oxygen saturation of the medium c_L,O2_^*^ and the oxygen transfer rate (OTR) in the shake flask. The conditions at the start of the experiment are ideally measured or calculated directly during inoculation. The maximum oxygen saturation in the medium depends on its composition as well as the incubator atmosphere and can be determined by a blank measurement and subsequently included in the calculation or adjusted by setting the dissolved oxygen concentration to 100% after saturation with air has been achieved. To calculate $C_{O_{2}}^{*}$, the atmospheric pressure p, the molar fraction of oxygen $x_{O_{2}}$ (which is lowered in CO_2_ incubators) and the temperature-dependent Henry constant must be known:

(1) $$C_{O_{2}}^{*}=p\cdot x_{O_{2}}\cdot H\left(T\right)$$

Several approaches exist for determining OTRs in shake flasks: existing mathematical approximations [37], measurement [50,51] or computational fluid dynamics (CFD) [52,53]. If the volumetric mass transfer coefficient k_L_a is known, the OTR can be calculated as follows:

(2) $$OTR=k_{L}a\cdot \left(C_{O_{2}}^{*}-C_{O_{2}}\right)$$

Provided that the system is in a state of equilibrium, the OTR is equal to the oxygen uptake rate (OUR), which is the product of the biomass concentration $C_{x}$ and $q_{O_{2}}$:

(3) $$q_{O_{2}}\cdot C_{X}=k_{L}a\cdot \left(C_{O_{2}}^{*}-C_{O_{2}}\right)\;\mathrm{OUR}=\mathrm{OTR}$$

Thus, $q_{O_{2}}$ can be calculated if the OTR and C_X_ are known. As q_O2_ is assumed to be constant during the exponential growth phase, C_X_ is proportional to the OTR and hence can be calculated if $C_{x,0}$ is known. The biomass concentration at timepoint t $C_{x\left(t\right)}$ as well as $\mu_{\max}$ can, therefore, be estimated by a curve fitting to Equation (4). This is possible using offline biomass measurements, OUR data or biomass concentrations from backscattered light measurements.

(4) $$C_{X}\left(t\right)=C_{X,0}\cdot e^{\mu_{\max}\cdot \left(t-t_{0}\right)}$$

Often-used yield coefficients compare the substrate used ($C_{S}$) with biomass ($Y_{X/S}$) or product ($Y_{P/S}$) formation but require metabolite measurements or well-trained models to do so.

(5) $$Y_{X/S}=\frac{C_{X,t}}{C_{S,t}}/Y_{P/S}=\frac{C_{P,t}}{C_{S,t}}$$

The specific growth rates determined online were compared and validated against those calculated offline. Since q_O2_ could only be determined using online data, literature data were used as a reference.

#### 2.2.1. Bacteria—*E. coli*

**Figure 2:**
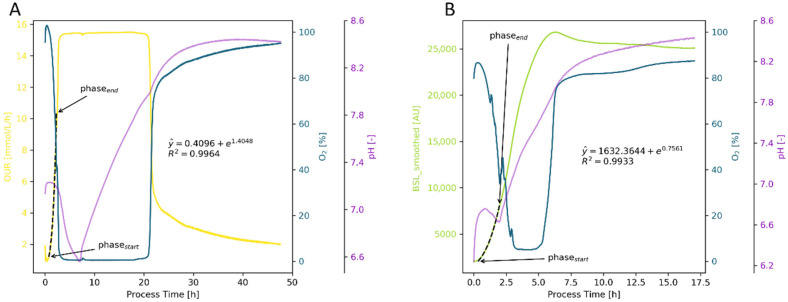
Evaluation of OUR and BSL for *E. coli* W3310 in TB (**A**) and LB (**B**) media. The OUR signal (**A**, yellow line) in unbaffled 500 mL shake flasks, cultivated with 50 mL filling volume, 180 rpm shaking speed and 50 mm shaking amplitude, gives a good estimation of the growth rate with µ_OUR, auto._ = 1.4048 h^−1^. The oxygen limitation after 4 h is clearly visible in the OUR and the O_2_ signal (blue line) until the glycerin has been completely consumed, which is indicated by a sharp increase in oxygen (21 h). The BSL signal (green line) in an experiment using LB (**B**) provides a good qualitative representation of the cultivation. However, the estimated growth rate µ_BSL, auto._ = 0.7561 h^−1^ is significantly lower compared with the growth rates estimated offline (µ_CDW, offline_ = 1.3237 h^−1^, Appendix B) and online based on the OUR (µ_OUR, auto._ = 1.2075 h^−1^, data not shown).

In the first experiments carried out with *E. coli* W3310 in complex media, it was found that opening the shaking incubator as well as sampling directly from the shake flask significantly influenced the measurement result, and subsequently, determination of the KPIs was no longer possible. Therefore, it was determined for all experiments that only at the beginning and end of the shaking would the incubator be opened and, if necessary, a sample taken. Various settings were tested for the different cultivation conditions, and two example results are shown in Figure 2. Cultivations in TB medium showed that the automated growth rate determined by the OUR led to good results (Figure 2A), with µ_OUR, auto._ = 1.4048 h^−1^ compared with the manually determined values from online (µ_OUR, man._ = 1.4201 h^−1^) and offline data (µ_CDW, offline_ = 1.3237 h^−1^). The exponential growth phase ended after approximately 5 h due to oxygen restriction and passed into a phase of slow, oxygen limited growth which lasted for 16 h. This phase was characterized by a plateau in the OUR. The end of the experiment was visible due to a sharp increase in oxygen and a drop in the OUR signal. This indicated the depletion of nutrients, in particular of glycerin and the consumable components of the complex media. 

Utilizing the BSL signal was, in general, less successful. Although a qualitative growth curve could be determined, the estimated values were too low. An example is depicted in Figure 2B, where the estimated growth rate using the BSL signal (µ_BSL, auto._ = 0.7561 h^−1^) is significantly lower than the OUR (µ_OUR, auto._ = 1.2075 h^−1^) or offline (µ_CDW, offline_ = 1.3237 h^−1^) based values. Additionally, in other experiments, OUR proved to outperform the BSL signal irrespective of whether baffles were used or not.

The calculation of growth rates from the OUR was successful for both complex media. For the LB experiments (six flasks), a growth rate of µ_CDW, offline_ = 1.2982 ± 0.1153 h^−1^ was determined offline, which agreed well with the values calculated manually (µ_man., online_ = 1.2752 ± 0.2049 h^−1^) and automatically (µ_auto., online_ = 1.2075 ± 0.1511 h^−1^) from online data. Similar values were obtained in the experiments in TB medium (6 flasks). With offline data, a growth rate of µ_CDW, offline_ = 1.2889 ± 0.0453 h^−1^ was estimated, compared with µ_man., online_ = 1.3421 ± 0.0419 h^−1^ with manually and µ_auto., online_ = 1.3778 ± 0.0191 h^−1^ with automatically determined online data. The TB experiments had a biomass yield of Y_X/S_ = 1.679 ± 0.032 g_CDW_ g_Gly_^−1^, whereby a CDW_max_ of 9.11 ± 0.21 g L^−1^ was achieved. For the experiments in LB medium, no yield was estimated as no carbon source was added. The maximum biomass concentration was 1.33 ± 0.10 g L^−1^ just from the complex media ingredients.

To investigate the influence of the media components, experiments were carried out with a chemically defined medium according to Biener, in addition to the complex media listed so far. The most striking differences were the coloration (clear instead of brown) and the lower variability of the media components. Interestingly, the clear color did not prove advantageous for BSL measurements in the low OD_600_ range. On the contrary, the growth rates calculated in this way were unrealistic, which could possibly be explained by low absorption and the associated increased reflection of the light from the liquid surface that acts as a mirror. The defined media components, on the other hand, ensured higher reproducibility, which was evident in the lower standard deviations. All determined growth rates were significantly lower compared with the complex media. With offline measurements, a growth rate µ_CDW, offline_ of 0.620 ± 0.019 h^−1^ was determined. The manually determined growth rate based on online data was slightly higher (µ_man., online_ = 0.6432 ± 0.004 h^−1^), and the algorithm-based rate was slightly lower (µ_auto., online_ = 0.582 ± 0.020 h^−1^). The biomass yield (Y_X/S_ = 0.434 ± 0.065 g_CDW_ g_Glc_^−1^) and the maximum biomass concentration CDW_max_ = 3.51 ± 1.27 g L^−1^ were significantly lower. This can be explained by the missing energy source from the complex media components and the absence of pH regulation, which led to an acidic regime as a result of acetate production. The specific oxygen consumption rate q_O2_ in the Biener medium was 2.132 ± 0.413 × 10^−2^ mol g^−1^ h^−1^, which agreed with reported data for *E. coli* in chemically defined media. Andersen and von Meyenburg reported a q_O2_ of 1.98 ± 0.17 × 10^−2^ mol g^−1^ h^−1^ in minimal medium with 2 g L^−1^ glucose during the exponential growth phase [54]. Lin et al. estimated a q_O2_ of 2 ± 0.2 × 10^−2^ mol g^−1^ h^−1^ with the same strain in chemically defined Teich media in a batch phase process [55]. Further data on experiments with *E. coli* can be found in Appendix B.

#### 2.2.2. Yeast—*S. cerevisiae*

**Figure 3:**
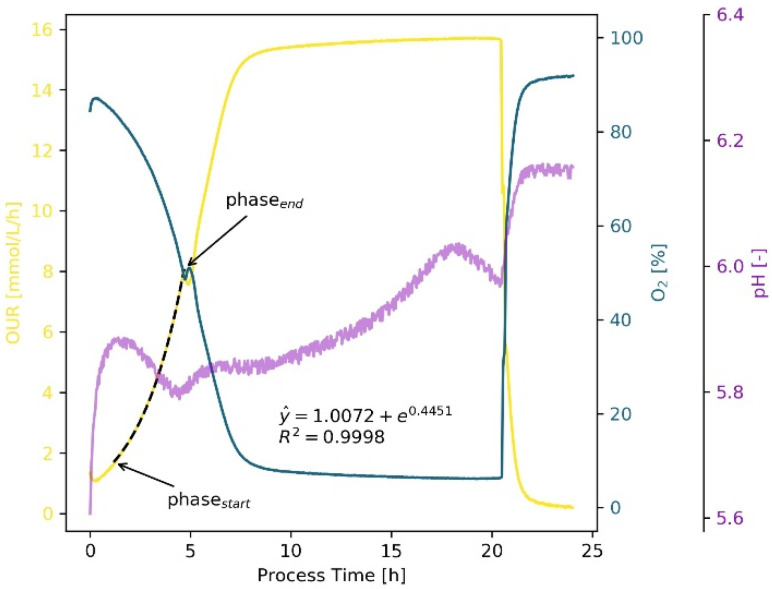
Cultivation of *S. cerevisiae* H1022 in YPD medium (250 mL unbaffled shake flask, 40 mL working volume, 180 rpm, 50 mm amplitude, 30 °C). The OUR curve (yellow) is used to determine the exponential growth phase, starting after a short (approx. 1.5 h) lag/adaption phase until shortly before the metabolic shift (5 h) from glucose consumption and ethanol formation to ethanol consumption. The estimated growth rate based on OUR during glucose consumption µ_OUR, auto._ was 0.445 h^−1^. Changes in metabolism are also visible as turning points in the pH curve (violet). Furthermore, oxygen limitation is visible by means of the constant OUR and O_2_ (blue) curves between 8 and 21 h. The consumption of all C-sources (glucose and ethanol) and thus the end of the growth phase is visible in all signals at 21 h (rise of O_2_ and pH, drop of OUR).

The basic design of the *S. cerevisiae* experiments corresponded to those with *E. coli*, but with higher working volumes. Of particular interest in these yeast cultivations were changes in metabolism that were visible online and were associated with different cultivation phases (Figure 3). After a brief adaptation period, the yeast grew exponentially, consuming glucose, and producing ethanol (between start and 5 h). The highest growth rate was expected during this phase. Thus, the algorithm cut off the adaptation phase and used the short increase in oxygen to set the phase starting and ending points. This shift from glucose consumption and ethanol formation to ethanol consumption was also visible in the pH, which instead of decreasing, started to increase. During ethanol consumption, the suspension was oxygen limited (in this example, between 8 and 21 h) and thus, the informative value of oxygen and OUR data decreased. After 18 h, a change in pH was visible, which may indicate the switch from ethanol to acetate or complex media components as energy sources. Finally, after 21 h, oxygen saturation and pH increased sharply while the OUR dropped, indicating a complete depletion of energy sources and the end of the cultivation. The online calculated growth rate of µ_OUR, auto._ = 0.445 h^−1^ fit very well to the manually determined ones using online data (µ_OUR, man._ = 0.454 h^−1^ and µ_CDW, offline_ = 0.453 h^−1^). Furthermore, with the online measurements, the q_O2_ was determined to be 3.12 mmol g^−1^ h^−1^, which was comparable to the literature results with *S. cerevisiae* [56].

**Figure 4:**
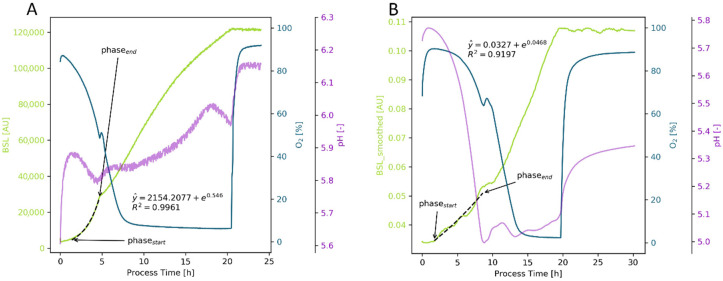
Comparison of backscattered light signals and growth rate estimations of *S. cerevisiae* H1022 in unbaffled 250 mL (**A**) and 500 mL flasks (**B**), cultivated at 30 °C in YPD medium. The BSL signal (light green) in the 250 mL flask (**A**) could be used without a filter and provided a fairly good result (µ_BS, auto._ is 0.546 h^−1^). However, using the BSL signal in the 500 mL flask (**B**), even after smoothing the signal, was not sufficient to obtain a realistic calculation of the growth rate (neither manually nor algorithm-based).

The growth rate estimation using the online backscattered light signal was less successful and associated with greater deviations (Figure 4). For the above-described experiment, a µ_BSL, auto._ of 0.546 h^−1^ was calculated. Even the manual determination using the backscattered light signal resulted in a comparatively large deviation (µ_BSL, man._ = 0.511 h^−1^), indicating that this method is not suitable for accurately and reliably determining KPIs.

The KPIs for *S. cerevisiae* were determined in 10 shake flask experiments under different cultivation conditions (however, always at 30 °C and with YPD), resulting in growth rates of µ_CDW, offline_ = 0.496 ± 0.034 h^−1^, µ_OUR, man._ = 0.489 ± 0.045 h^−1^ and µ_OUR, auto_ = 0.474 ± 0.042 h^−1^, with a biomass yield, Y_X/S_, of 0.812 ± 0.168 g_CDW_ g_Glc_^−1^ and a q_O2_ = 2.588 ± 0.392 × 10^−3^ mol g^−1^ h^−1^. The relatively large standard deviation in the biomass yield can be explained by metabolic differences in baffled and unbaffled flasks. The latter reach oxygen limitation earlier, increasing the total cultivation time and requiring more energy for maintenance metabolism. This was also visible in the maximum achieved biomass. In baffled flasks, CDW_max_ was 9.4 g L^−1^, whereas in unbaffled flasks, CDW_max_ was 7.7 g L^−1^. The type of flask had no influence on the maximum growth rate in the batch experiments performed, since exponential growth had already been achieved before oxygen limitation occurred.

#### 2.2.3. Plant Cells—*Vitis vinifera*

**Figure 5:**
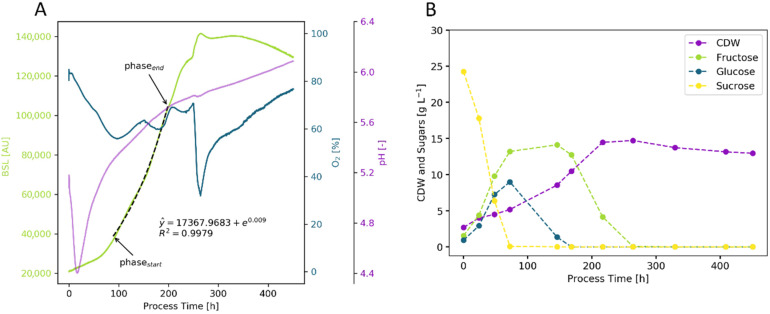
Cultivation of *V. vinifera* in 500 mL unbaffled shake flasks with 100 mL working volume, 120 rpm shaking rate and 50 mm shaking amplitude. For the algorithm-based determination of the growth rate, the BSL signal was used (**A**, light green line, µ_BSL, auto._ = 0.0090 h^−1^). Changes in carbon source uptake (**B**, sucrose, glucose, and fructose measurements) are visible as drops in the dissolved oxygen signal (**A**, blue line). The uptake of inorganic ions (data not shown) is visible at the pH signal (purple line), primarily the turning point after 16 h.

The cultivation of plant cells had some major differences compared with the microbial fermentations considered so far. First, they grew much more slowly, with doubling times of three days or more. This was accompanied by correspondingly long cultivation times, usually two weeks for *V. vinifera* batch cultivations. At the same time, very high cell densities could be achieved, so that up to 80% of the suspension could consist of cells. This was associated with a significant increase in viscosity. The metabolism of sucrose could be regarded as a metabolic peculiarity, which was first enzymatically and extracellularly cleaved into glucose and fructose. Subsequent use occurred simultaneously, with glucose being preferentially consumed. These metabolic changes were also very clearly visible in the online signals (Figure 5). After the sucrose had been completely cleaved and the cells had completed their adaptation phase (72 h), there was a brief increase in the O_2_ signal. A short adaptation in the O_2_ signal was also seen when the glucose had been completely consumed (168 h). Particularly striking was the strong decrease in the dissolved oxygen concentration after 264 h, the time at which the last carbon source, fructose, was consumed.

This illustrates the advantages and disadvantages of measuring the oxygen in plant cell shake flask cultivations. This, and corresponding knowledge of plant physiology, allowed substrate consumption to be followed online without having to wait for time-consuming substrate measurements such as HPLC. However, these “jumps” in the oxygen signal caused deviations in the OUR calculated from it, making it unsuitable for calculating the growth rate. 

In this case, the size of the plant cells and the high cell density within the suspension proved to be an advantage. The BSL signal was clear and unnoisy throughout the cultivation period and correlated very well to the cell dry weight concentration, even in the death phase (Figure 5). The algorithm-based growth rate calculated from this (µ_BSL, auto._ = 0.0090 h^−1^) was also very similar to the rates calculated manually (µ_BSL, man._ = 0.0093 h^−1^) and offline (µ_CDW,offline_ = 0.0090 h^−1^). The pH signal could be used to interpret the use of inorganic metabolites. The preferential uptake of ammonium and phosphate in the adaptation phase was accompanied by a strong decrease in pH. During the subsequent uptake of nitrate, the pH increased continuously until the end of cultivation (Figure 5A).

The KPIs determined from all the experiments (12 flasks) can be summed up as follows: The growth rates calculated using µ_BSL, auto._ = 0.0080 ± 0.001 h^−1^, µ_BSL, man._ = 0.0088 ± 0.001 h^−1^ and µ_CDW, offline_ = 0.0084 ± 0.001 h^−1^ matched well, with a slight underestimation of the manually calculated growth rates based on the BSL signal and a slight overestimation when manually estimating them. The biomass yield Y_X/S_ was 0.4744 ± 0.0366 g_CDW_ g_Glc_^−1^, resulting in maximum cell dry weight concentrations of CDW_max_ = 15.298 ± 0.881 g L^−1^. The specific oxygen consumption rate q_O2_ was 2.293 ± 0.400 × 10^−4^ mol g^−1^ h^−1^, compared with q_O2_ values from 1.1 to 7.1 × 10^−4^ mol g^−1^ h^−1^ in *V. vinifera* L. cv Gamay suspension cultures in Gamborg B5 medium, reported by Pépin et al. [57].

#### 2.2.4. Animal Cells

**Figure 6:**
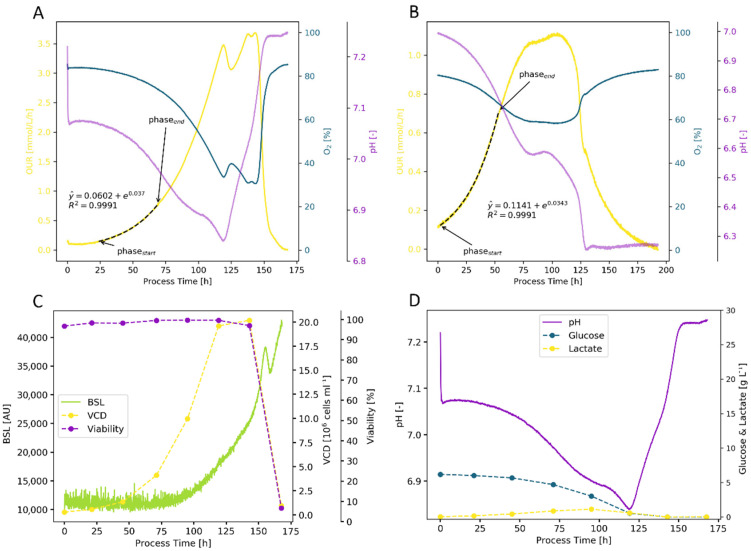
Cultivation of ExpiCHO-S 6H8 (**A**,**C**,**D**) and CHP DP12 #1934 (**B**) cells in unbaffled 250 mL Erlenmeyer flasks with 80 mL working volume, 120 rpm shaking rate and 50 mm shaking amplitude. Growth rate determination was performed with the OUR signal (yellow line), resulting in µ_OUR, auto._ = 0.037 h^−1^ for ExpiCHO-S (**A**), and µ_OUR, auto._ = 0.0343 h^−1^ for CHO DP-12 (**B**). For low cell densities, the BSL signal had a high signal to noise ratio, which decreased as cell density (**C**) increased. The BSL signal increased as viability decreased, indicating a measurement of the dead cell density (**C**). The production and consumption of lactate was visible in the pH signal (purple line), with the lactate shift at 120 h clearly visible (**D**).

The study of animal cell cultures was performed in several steps. First, the two CHO cell lines were characterized (Figure 6). Similar to the microbial cultivations, the OUR signal provided a very good estimation of the specific growth rate. Due to the relatively high filling volume in combination with the lower shaking rates and doubling times in the range of 18 to 24 h, the signal was not noisy and was easy to interpret Figure 6). For the ExpiCHO-S 6H8 cell line, the µ_OUR, auto._ = 0.0370 h^−1^ was similar to the µ_OUR, man._ = 0.0365 h^−1^ and both were slightly lower than the µ_VCD, offline_ with 0.0389 h^−1^ (Figure 6A). For CHO DP12 #1934, the results were all similar, with µ_OUR, auto._ = 0.0340 h^−1^, µ_OUR, man._ = 0.0347 h^−1^, and µ_VCD, offline_ = 0.0348 h^−1^ (Figure 6B). The pH signal was very interesting too, as it indicated whether the metabolic shift from lactate formation to consumption was successful or not. The increase in pH after 120 h for the ExpiCHO-S cells (Figure 6A) correlated to the uptake of lactate and thus total consumption of the initial glucose, resulting in higher yields compared with an unsuccessful lactate shift with no pH change at the end in the depicted CHO-DP12 cultivation (Figure 6B). The BSL signal showed a surprising trend for animal cell cultures. For the first 70 h, there was no change, despite cell growth. However, as viability decreased, the BSL signal increased. For animal cell cultures, this resulted in a correlation of the BSL signal with dead cell density. This could be explained by a change in the cell surface during death, which was also visually indicated by a milky turbidity in the cell suspension during the death phase.

The growth rates based on OUR for ExpiCHO-S (6 flasks) again showed good agreement with µ_VCD, offline_ = 0.0390 ± 0.007 h^−1^, µ_OUR, man._ = 0.0359 ± 0.005 h^−1^, and µ_OUR, auto_ = 0.0362 ± 0.007 h^−1^. Comparable results were also obtained with the CHO DP-12 cells (eight flasks), resulting in growth rates of µ_VCD, offline_ = 0.0349 ± 0.017 h^−1^, µ_OUR, man._ = 0.0335 ± 0.018 h^−1^, and µ_OUR, auto_ = 0.0327 ± 0.011 h^−1^. For both CHO cell lines, the OUR-based growth rates were slightly lower than the offline data-based values. The standard deviation was minimal, indicating that with standardized inoculum preparation and chemically defined media, high reproducibility would be achievable. More significantly than growth rates, the cells differed in yield, with Y_X/S_ = 3.126 ± 0.065 × 10^6^ cells g_Glc_^−1^ for ExpiCHO-S and Y_X/S_ = 1.990 ± 0.140 × 10^6^ cells g_Glc_^−1^ for CHO DP-12. The lower cell specific oxygen consumption rate for ExpiCHO-S (q_O2_ = 2.283 ± 0.238 × 10^−13^ mol cell^−1^ h^−1^) compared with CHO DP-12 cells (q_O2_ = 2.884 ± 0.623 × 10^−13^ mol cell^−1^ h^−1^) indicated that ExpiCHO-S cells seem to have a more effective metabolism. This was also visible in the product yield, which was double for ExpiCHO-S (Y_P/S_ = 0.0575 ± 0.0055 g_IgG_ g_Glc_^−1^) compared with CHO DP-12 (Y_P/S_ = 0.0267 ± 0.0038 g_IgG_ g_Glc_^−1^). The q_O2_ values were in good agreement with the literature values (3.0 to 3.2 × 10^−13^ mol cell^−1^ h^−1^ for CHO DP-12 [58] and 2.3 × 10^−13^ mol cell^−1^ h^−1^ for CHO-S cells [59]). Additional data on the CHO DP-12 and as well as the characterization of HEK and High Five cultivations are provided in Appendix B. 

### 2.3. Evaluation of Measurement Techniques

No single measurement technique was suitable for all of the organism-flask combinations, but at least one was found to be appropriate in each examined case (Table 2). In general, the OUR was more diverse in its use and may be the signal of choice for all culture types except plant cells. However, there were some drawbacks. First, the k_L_a-value must be known and an initial oxygen saturation until equilibrium must be performed, as even slight variations (>1% difference in oxygen saturation) have significant effects on the growth rate calculation. Second, if the suspension reaches oxygen limitation, no information gain is possible. Finally, signal disturbances, whether resulting from metabolic changes or from disturbing the shaking platform, may render the automated signal interpretation useless. Thus, it is recommended not to open the shaker and especially not to sample directly from the measurement flasks. Furthermore, the change in the shaker atmosphere and temperature may also have a significant influence on the signal. 

The BSL signal was not affected by oxygen limitation and the interruption caused by a stopped shaker or a removed flask was less severe. Thus, the signal is relatively robust, as long as the culture is dense enough, which is the case for plant cell cultures or higher density microbial cultivations. However, baffled flasks and the bubbles they introduce into the suspension will result in a higher signal to noise ratio, especially for low filling volumes and high shaking rates. Interestingly, changes in size and surface affect the BSL signal, resulting in the detection of mainly dead cells in animal cell cultivations. 

The pH wa an excellent supporting parameter, indicating metabolic activities, e.g., the switch from glucose consumption and acetate production to acetate consumption or the metabolic shift from lactate formation to consumption for CHO cells. Furthermore, these metabolic changes were still visible, even if the oxygen signal could not be used due to oxygen limiting conditions.

### 2.4. Evaluation of KPIs

To evaluate the KPIs, the algorithm-based online data were compared with manually calculated online and offline data. All manual calculations were performed by the same user to reduce any human-related bias which may occur if several users manually set the exponential growth phases. 

As shown in the previous subsections, the OUR can be used for most organisms and is essential for the calculation of the q_O2_. The BSL signal does not correlate linearly to the OD_600_, which is probably the reason for the lower accuracy of this method for growth rate estimations. Calibration allows the BSL signal to be converted directly to OD_600_ or CDW, increasing accuracy. However, this involves further manual work, which contradicts the approach presented here. Manually and automatically determined growth rates based on the OUR and the BSL data are compared in Table 3. Very good agreement between the growth rates determined offline and by means of the algorithm is shown, with an average deviation of 6.5%. The highest deviation of 12.8% was found for the HEK cells, and the deviation was less than 7% for all other cells. 

The obtained online data could be confirmed by the measured offline data. However, an additional verification, especially for the specific oxygen uptake rates obtained only by online data, resulted from a comparison with already published values (Table 4). This comparison shows both the accuracy and the advantages of the proposed approach. Provided that comparable experiments (i.e., strain, medium, and process conditions similar) are available, the determined values of the presented approach compare very well with them.

However, as soon as the selected parameters deviated more strongly from the reference data, the range of literature values was also significantly larger, as can be seen, for example, in the growth rate of High Five cells or the specific oxygen uptake rate of *S. cerevisiae*. Another problem arose when complex media components were used, which made it very difficult to compare the maximum cell number or yield for media such as LB, TB and YPD. It was equally difficult to determine the biomass yield of High Five and HEK293 cells in a batch process without infection, as most publications are dedicated to the much more challenging production fed-batch processes with virus infection. Therefore, for the characterization of a new cell–medium combination or when changing the process conditions, the experimental determination of the KPI is recommended, which can be performed easily, automatically, and precisely with the presented approach.

## 3. Conclusions and Outlook

In contrast to state-of-the-art approaches, where media and strains are laboriously characterized or optimized using manually and invasively determined KPIs, the presented, novel workflow can determine KPIs automatically and standardize them in early-stage bioprocess development, as described in Section 2.1. By combining data science methods, adjusted to the small amount of data usually available in early-stage development, and measurements of online, non-invasive sensors, it has been shown that KPIs, which are comparable to results from the literature and manual evaluation, can be efficiently extracted from shake flask experiments. Furthermore, it has also been shown that the discussed workflow provides solid and robust results under realistic conditions. 

The experiments carried out to evaluate the performance of the algorithm were conducted by different operators, over different time periods, with different flasks, and with different media and organisms, providing a good representation of the expected reality in bioprocess development. In Section 2.2, we have shown that the developed workflow can identify the exponential growth phase for the performed experiments just as well as an experienced user can using manual evaluation, see Table 3. Clearly, the accuracy of the algorithm depends on the quality of the online signal that is used, but the phase optimization technique using the algorithm and the recipe database substantially contribute to increasing the robustness of the workflow. Therefore, we conclude that the application of this algorithm in research and industry can help save time and resources for strain and medium characterization and optimization.

The robustness of phase detection could be improved by using further online signals such as dissolved CO_2_ in addition to pH, dissolved oxygen, and OUR or BSL, as these data parameters also provide further insight into the growth behavior of the observed organism. Further experiments should verify the application of challenging cultivation conditions. This includes phototrophically growing organisms such as algae or fungal and bacterial cultures, which have a mycelial growth, e.g., used for antibiotic production, and are preferably cultivated on a growth surface but can also grow on the measuring surface of the sensor spots, and pose a challenge when cultivating in shake flasks [80].

## 4. Materials and Methods

### 4.1. General Equipment and Online Measurement Systems

**Figure 7:**
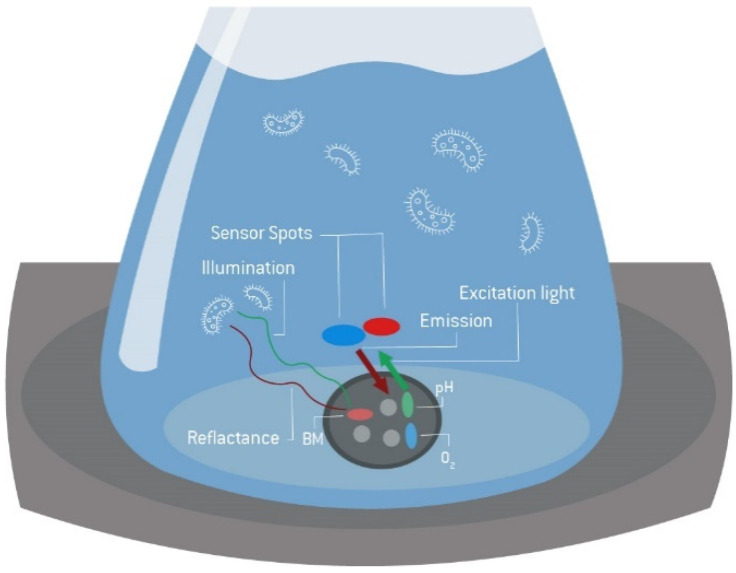
Measurement principle of the SFR vario. The biomass is measured via backscattered light, pH, and O_2,_ via integrated sensor spots. Backscattered light is measured using an LED with peak wavelength of about 605 nm and the reflected light is detected by a photodiode.

The experiments were performed in 250 or 500 mL disposable Erlenmeyer flasks (Corning Inc., New York, NY, US) with and without baffles in Multitron Pro, Multitron Cell (Infors HT, Bottmingen, CH) and LT-X (Adolf Kühner AG, Birsfelden, CH) shaking incubators with 25 or 50 mm shaking amplitudes. Online measurements of backscattered light, dissolved oxygen concentration, and pH were carried out using up to four SFR vario devices (PreSens, Regensburg, DE) and corresponding disposable shake flasks equipped with sensor spots (Figure 7). Both sensor spots contain reversible fluorescent dyes that indicate changes in O_2_ and pH. In contrast to electrodes, these sensors do not require calibration as they come pre-calibrated. Calibration constants are valid for a certain production cycle of flasks, indicated by a batch number. The device is placed inside the shaker and has integrated rechargeable batteries. The controlling PC establishes a wireless Bluetooth connection to initiate the readouts at flexible time intervals. To avoid disturbing the online measurements by stopping the shaker and removing the shake flasks, all comparative offline measurements (except for the determination of initial cell density and substrate concentration, which was performed in each shake flask) were conducted in reference flasks.

The PreSens system was chosen to establish the workflow because the simultaneous measurement of dissolved oxygen, biomass signal via backscattered light, and the calculated oxygen uptake rate allowed cross-validation of the data obtained. However, the approach can also be performed with other systems described in Section 1, such as the RAMOS, the Kuhner TOM or the CGQ. Additionally, of interest is the combination of different measurement systems to ensure a comparable verification, as demonstrated by Anderlei et al. [81].

### 4.2. Cultivation Details

#### 4.2.1. Bacteria Cultivation—*E. coli*

The experiments were performed using the W3110 *E. coli* strain (thyA3662supOλ−, DSMZ ordering number: 5911) and three media:

- Lysogeny broth (LB) medium with 5 g L^−1^ yeast extract (cat. Y1625, Sigma-Aldrich, St. Louis, MI, USA), 10 g L^−1^ tryptone (cat. 95039, Sigma-Aldrich) and 5 g L^−1^ sodium chloride (cat. S9888, Sigma-Aldrich) [82];
- Terrific broth (TB) medium with 24 g L^−1^ yeast extract (cat. Y1625, Sigma-Aldrich), 20 g L^−1^ tryptone (cat. 95039, Sigma-Aldrich), 4 mL L^−1^ glycerin (cat. 49770, Sigma-Aldrich) and a phosphate buffer consisting of 0.17 mol L^−1^ KH_2_PO_4_ (cat. P5655, Sigma-Aldrich) and 0.72 mol L^−1^ K_2_HPO_4_ (cat. P3786, Sigma-Aldrich) [83];
- Modified Biener medium consisting of glucose (10 g L^−1^), a mineral salt solution, a trace element solution and MgSO_4_ solution [60]. Details can be found in Appendix A.

The two complex media LB and TB have the same complex components, but TB is richer, has an additional carbon source (glycerin), and is buffered. The LB was mixed completely and autoclaved, while the TB media and buffer were autoclaved separately and mixed after the components had cooled down. The chemically defined media according to Biener et al. [60] with reduced glucose concentrations compared with the original recipe, consisted of different salt and trace element stock solutions as well as glucose and MgSO_4_ solutions (Appendix A). All stock solutions were autoclaved separately and mixed after cooling down. Accordingly, the influence of different media (complex vs. defined), different temperatures and different shaking conditions were tested. For biomass quantification, the optical density at 600 nm (OD_600_) was measured using a SmartSpec Plus photometer (Bio-Rad, Hercules, CA, US) and the cell dry weight (CDW) was determined gravimetrically in 1.5 mL tubes. Metabolites were analyzed using a Cedex Bio (Roche Diagnostics, Mannheim, Germany) and the corresponding kits for glucose, ammonia, and phosphate.

#### 4.2.2. Yeast Cultivation—*S. cerevisiae*

*S. cerevisiae* H1022 (ATCC cat. 32167), a Crabtree-positive yeast, was selected as the second microbial representative to monitor the metabolic switch between glucose uptake and ethanol formation to ethanol consumption online. For these cultivations, the peptone, dextrose, yeast extract (YPD) complex medium was used [84], composed of 10 g L^−1^ yeast extract (cat. Y1625, Sigma-Aldrich), 20 g L^−1^ tryptone (cat. 70173, Sigma-Aldrich) and 20 g L^−1^ glucose (cat. G2870, Sigma-Aldrich), which were autoclaved together.

Temperature was set to 37 °C, shaking frequency to 180 rpm, and shaking amplitude to 50 mm. Biomass was determined identically to the *E. coli* experiments. For metabolite analytics, ethanol was measured instead of ammonia.

#### 4.2.3. Plant Suspension Cultivation—*Vitis vinifera*

As a model plant cell suspension culture, *Vitis vinifera* cv. Uva italia was selected. The cell line was established by Silvie Cuperus in 2003 [67] and maintained as calli (25 °C, without light, subcultivation every 4 weeks). The suspension culture was cultivated in a modified Murashige and Skoog [85] medium (cat. M0222, Duchefa Biochemie B.V., Haarlem, Netherlands) with 30 g L^−1^ sucrose (cat. 4621.5, Roth AG, Arlesheim, Switzerland), 0.25 g L^−1^ casein hydrolysate (cat. C1301, Duchefa), 1 µmol L^−1^ kinetin (cat. K3378, Sigma-Aldrich) and 1-Naphthaleneacetic acid (NAA, cat. N1641, Sigma-Aldrich) each. All components were mixed, the pH was adjusted to 5.8 using 1 mol L^−1^ KOH (cat. 1091081000, Merck KGaA, Darmstadt, Germany) and the media was sterilized by filtration (VacuCap, cat. 4622, Pall, Port Washington, NY, USA). Biomass analytics included the measurement of packed cell volume as well as fresh and dry biomass. Electrical conductivity and pH were determined from the supernatant by probes (FiveEasy, Mettler Toledo, Greifensee, Switzerland). Metabolic analytics for sucrose, glucose, fructose, phosphate, ammonia, and nitrate in the supernatant were performed by HPLC (Shimadzu Deutschland GmbH, Duisburg, Germany) with a 940 Professional Vario IC (Metrohm Schweiz AG, Zofingen, Switzerland).

#### 4.2.4. Animal Cell Cultivation—CHO, High Five, HEK293

Two Chinese hamster ovary (CHO) cell lines, one human embryo kidney (HEK) 293 and one insect cell line (*Trichoplusia ni* BTI-Tn-5B1-4, High Five) were used for the animal cell culture experiments (Table 5).

Both CHO cell lines produce the antibody immunoglobulin G (IgG) and behave in the same manner as industrial mammalian production cell lines. HEK cells are also widely used, but tend to form larger aggregates [86]. High Five insect cells have similar morphological characteristics to CHO cells but are cultured without CO_2_ based pH control and at lower temperatures. The applied cultivation conditions are summarized in Table 6. The effect of different clones, media and cultivation conditions should test the robustness of the methodology.

Cell density and viability were measured using a Cedex HiRes (Roche Custom Biotech) for the CHO and High Five cells, and with a NucleoCounter NC200 (Chemometec A/S, Allerod, Denmark) for the HEK293 cells. Metabolites (glucose, glutamine, ammonia), as well as the product IgG, were determined using a Cedex Bio (Roche Custom Biotech) and the corresponding analytic kits.

### 4.3. Software

The SFR varios that were used were controlled by the associated PreSens Flask Studio PFS software. The software connected to the device at each time point to initiate the measurement and retrieve the data that were stored in the integrated SQL database. Data were then visualized online as diagrams and could be compared with historical runs in a cumulative graph.

For more advanced analytics, as described in chapter 2.1, the data were transferred from the SQL database via a REST API to commercially available PAS-X Savvy 2022.03 software (Körber Pharma Software GmbH, Vienna, Austria). This additional interface was implemented in PFS. This software was used to develop the relevant algorithms and analyses for this work using Python 3.79 (Python Software Foundation, available online: https://www.python.org/, accessed on 22 March 2022).

## Figures and Tables

**Table 1:** Information stored in the recipe. The values of the individual attributes may differ from organism to organism, especially if the cultivation time varies considerably.

Attribute	Information	Value
O_2, threshold_	Oxygen limit when it can be assumed that exponential growth is impossible.	10–20%
Growth speed	Growth speed of the organism	Fast, medium, slow

**Table 2:** Organism-based evaluation of BSL and OUR signal for baffled and unbaffled shake flasks.

Organism	Flask Type	Backscatter	OUR
*E. coli*	Without baffles	Average	Good
	With baffles	Noisy at low filling volumes	Good
*S. cerevisiae*	Without baffles	Average	Good
	With baffles	Noisy at low filling volumes	Good
*V. vinifera*	With and without baffles	Good	Metabolic changes affect signal
CHO cells	Without baffles	Detection of dead cells	Good
High Five	Without baffles	Detection of dead cells	Good
HEK293	With and without baffles	Detection of dead cells	Good

**Table 3:** Overview of the KPIs for all investigated organisms under standard conditions described in Section 2. For every investigated organism–medium combination, the number of replicates is indicated in brackets (replicates). µ_offline_ was calculated based on measurements of cell dry weight for *E. coli*, *S. cerevisiae* and *V. vinifera* and viable cell density for CHO, HEK and High Five cells. The calculation of µ_BSL_ and µ_OUR_ was distinguished between manual (_man_) and automatic (_auto_) evaluation. The use of the BSL signal appeared to be useful only for *S. cerevisiae* and *V. vinifera*, whereas the use of the OUR only for *V. vinifera* did not seem to be purposeful. The calculated cell-specific oxygen uptake rates and biomass yields are based on the biomass measurements typically used for the organisms, i.e., cell dry weight for *E. coli*, *S. cerevisiae* and *V. vinifera*, and viable cell density for CHO, HEK and High Five.

Organism	Medium	µ_offline_	µ_BSL, man._	µ_BSL, auto._	µ_OUR, man._	µ_OUR, auto._	q_O2_	Y_X/S_
	(Replicates)	[h^−1^]	[h^−1^]	[h^−1^]	[h^−1^]	[h^−1^]	[mol g^−1^ h^−1^]	[g g^−1^]
*E. coli*	LB (6)	1.2982 ± 0.1153	-	-	1.2752 ± 0.2049	1.2075 ± 0.1511	1.65 ± 0.28 × 10^−2^	- ^1^
	TB (6)	1.2889 ± 0.0453	-	-	1.3421 ± 0.0419	1.3778 ± 0.0191	1.82 ± 0.33 × 10^−2^	1.679 ± 0.032 ^2^
	Biener (4)	0.6199 ± 0.0194	-	-	0.6432 ± 0.0041	0.5820 ± 0.0021	2.13 ± 0.41 × 10^−2^	0.434 ± 0.065
*S. cerevisiae*	YPD (10)	0.4964 ± 0.0344	0.6226 ± 0.1294	0.5110 ± 0.0907	0.4895 ± 0.0446	0.4744 ± 0.0424	2.59 ± 0.39 × 10^−3^	0.812 ± 0.168 ^2^
*V. vinifera*	MS (12)	0.0084 ± 0.0009	0.0088 ± 0.0009	0.0080 ± 0.0008	-	-	2.29 ± 0.40 × 10^−4^	0.474 ± 0.037
							**[mol cell^−1^ h^−1^]**	**[10^6^ cells g^−1^]**
CHO DP-12	TC-42 (8)	0.0349 ± 0.0017	-	-	0.0335 ± 0.0018	0.0327 ± 0.0011	2.88 ± 0.62 × 10^−13^	1.990 ± 0.140
ExpiCHO-S	SPM (6)	0.0390 ± 0.0007	-	-	0.0359 ± 0.0005	0.0362 ± 0.0007	2.28 ± 0.24 × 10^−13^	3.126 ± 0.065
High Five	Exp.Five (8)	0.0445 ± 0.0014	-	-	0.0453 ± 0.0017	0.0430 ± 0.0010	4.18 ± 0.02 × 10^−13^	1.274 ± 0.057
HEK293	FS293 (6)	0.0315 ± 0.0026	-	-	0.0365 ± 0.0010	0.0355 ± 0.0005	9.86 ± 3.76 × 10^−14^	1.392 ± 0.151

^1^ No yield was calculated as growth was only supported by complex media ingredients. ^2^ The yield for complex media TB and YPD was significantly higher because complex media components were used for maintenance metabolism and biomass growth in addition to the added carbon source.

**Table 4:** Comparison of automatically determined growth rates, specific oxygen uptake rates and biomass yield with published reference values. As far as possible, similar/same strains, media and process conditions were considered for the selection of literature data.

Organism	µ_auto._^1^	µ_lit_	q_O2, auto._	q_O2, lit._	Y_X/S_	Y_X/S,lit_	References
	[h^−1^]	[h^−1^]	[mol g^−1^ h^−1^]	[mol g^−1^ h^−1^]	[g g^−1^]	[g g^−1^]	
*E. coli* ^2^	0.5820 ± 0.0021	0.54-0.56	2.13 ± 0.41 × 10^−2^	1.3–2.2 × 10^−2^	0.434 ± 0.065	0.50–0.54	[54,55,60,61]
*S. cerevisiae*	0.4744 ± 0.0424	0.42–0.51	2.59 ± 0.39 × 10^−3^	1.0–9.0 × 10^−3^	0.812 ± 0.168	N.A. ^2^	[56,62,63,64,65,66]
*V. vinifera*	0.0080 ± 0.0008	0.0065–0.01	2.29 ± 0.40 × 10^−4^	1.1–7.1 × 10^−4^	0.474 ± 0.037	0.47–0.49	[57,67,68]
			**[mol cell^−1^ h^−1^]**	**[mol cell^−1^ h^−1^]**	**[10^6^ cells g^−1^]**	**[10^6^ cells g^−1^]**	
CHO DP-12	0.0327 ± 0.0011	0.0358–0.0363	2.88 ± 0.62 × 10^−13^	3.10 × 10^−13^	1.990 ± 0.140	2.063–2.216	[58,69,70,71,72]
ExpiCHO-S	0.0362 ± 0.0007	0.0316–0.0422	2.28 ± 0.24 × 10^−13^	2.30–2.90 × 10^−13^	3.126 ± 0.065	3.3–3.4	[59,73]
High Five	0.0430 ± 0.0010	0.028–0.044	4.18 ± 0.02 × 10^−13^	2.88–9.00 × 10^−13^	1.274 ± 0.057	1.250	[74,75,76]
HEK293	0.0355 ± 0.0005	0.03–0.05	9.86 ± 3.76 × 10^−14^	1.30–1.85 × 10^−13^	1.392 ± 0.151	N.A.	[77,78,79]

^1^ except *Vitis vinifera*, all µ_auto._ were based on OUR values. ^2^ Since the yields for LB and TB depend on the complex substrates used, the values for the chemically defined Biener medium were used. Therefore, a meaningful comparison with literature data was also not possible for the complex YPD medium.

**Table 5:** Overview of the cell lines (with origin or ordering number) and media (all serum-free or chemically defined).

Cell Line	Origin/Source	Media
CHO DP-12 #1934	Subclone, courtesy of Prof. Noll, Bielefeld	TC-42 (cat. 511-0001, Xell AG, Bielefeld, Germany) ^1^
ExpiCHO-S-6H8	Thermo Fisher Scientific, cat. A29127	ExpiCHO Stable production medium (cat. A3711001) ^2^
Freestyle 293 (HEK)	Thermo Fisher Scientific, cat. R79007	FreeStyle Expression 293 (cat. 12338018)
High Five	Thermo Fisher Scientific, cat. B85502	Express Five SFM (cat. 10486025) ^3^

With addition of: ^1^ 6 mmol L^−1^ Glutamine (cat. 25030081, Thermo Fisher Scientific, Waltham, MA, USA), 0.1 mg L^−1^ LONG^®^ R3 IGF-I human (cat. 60356-, SAFC Biosciences); ^2^ 4 mmol L^−1^ GlutaMAX (cat. 35050-061, Thermo Fisher Scientific); ^3^ 18 mmol L^−1^ L-Glutamine (cat. 25030081, Thermo Fisher Scientific).

**Table 6:** Cultivation conditions for the selected cell lines including shaker temperature, shaking rate, relative filling volume (V_rel_) and CO_2_ concentration in the incubator atmosphere. Humidity was set to 80% for all experiments.

Cell Line	Temperature [°C]	Shaking Rate [rpm]	V_rel_ [%]	CO_2_ [%]
CHO DP-12	37	120	32	7.5
EXPI-CHO S	37	130	24–32	8.0
Freestyle 293	37	130	32	8.0
High Five	27	100	24	0.0

**Figure A1:**
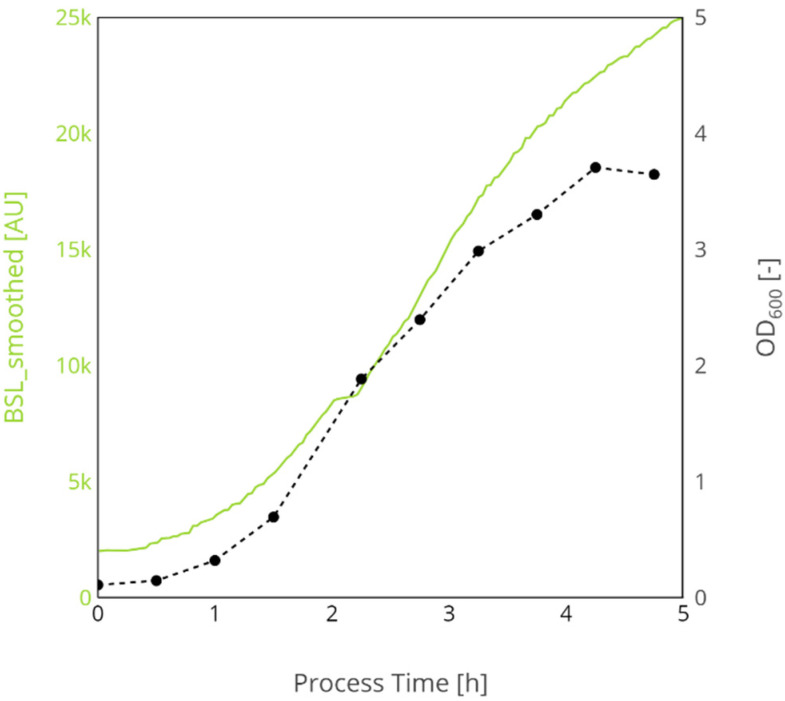
Offline growth data of *E. coli* W3110 in LB medium. The difference in increase between offline and online measured biomass concentration in the absence of calibration leads to significant differences in the calculated growth rate (µ_offline_ = 1.323 h**^−^**^1^ compared with µ_BSL, auto._ = 0.7561 h**^−^**^1^).

**Figure A2:**
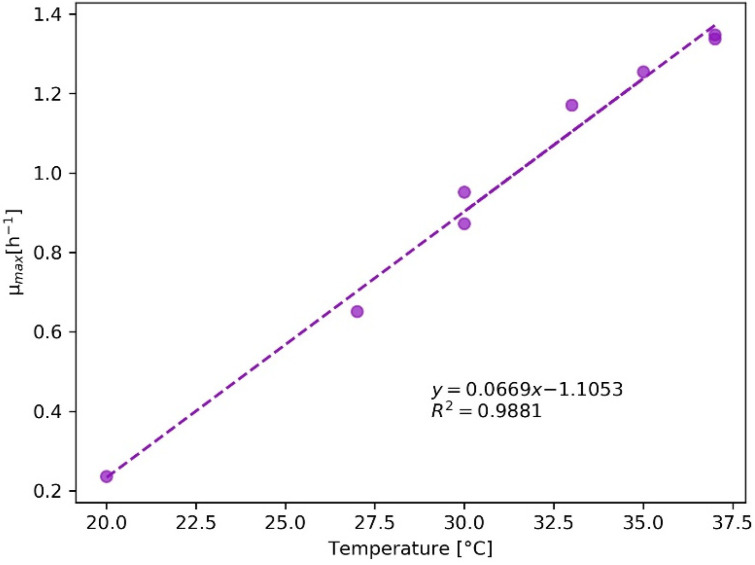
Dependence of growth rate on temperature in a *E. coli* cultivation (20 to 37 °C, 200 rpm, 25 mm shaking amplitude, 40 mL TB medium in 500 mL baffled shake flasks). The growth rates were calculated with online OUR data.

**Figure A3:**
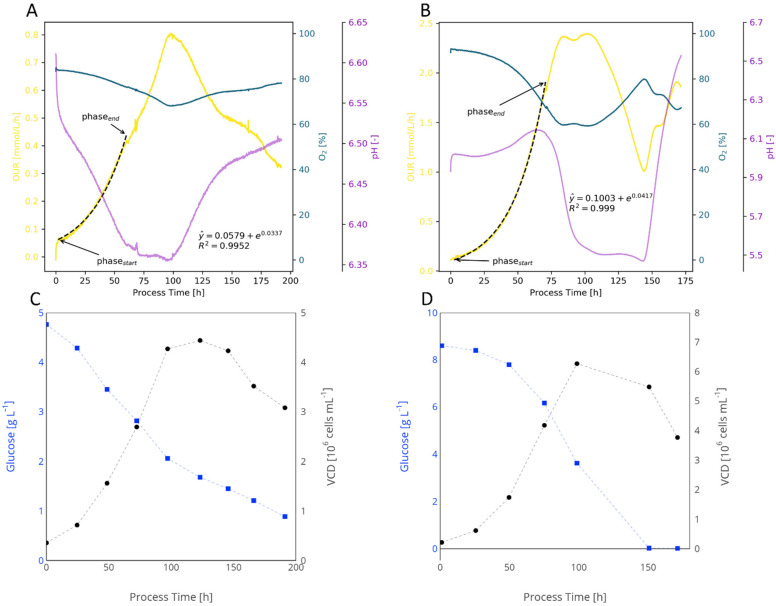
Evaluation of HEK cells in 250 mL shake flasks with 80 mL working volume (**A**,**C**) and High Five cells in 500 mL shake flasks with 100 mL working volume (**B**,**D**). Both cultivations were performed with a shaking rate of 130 rpm and a shaking diameter of 50 mm. The OUR signal (yellow line) was used for growth rate calculation, resulting in µ_OUR, auto._ = 0.00337 h^−1^ for HEK and µ_OUR, auto._ = 0.0417 h^−1^ for High Five cells. The metabolic shift from lactate formation to consumption for HEK cells was clearly visible in the pH signal (purple line, **A**).

## Data Availability

The data presented in this study are available on request from the corresponding author.

## Appendix A

### Recipe for 1 L E.coli Medium

This recipe uses a modified medium from Biener et al. [60] with lowered glucose concentration.

All stock solutions were mixed after autoclaving.

- 40 mL of glucose stock solution with 500 g L^−1^ glucose;
- 480 mL of mineral stock solution, consisting of:
  o 2.1 g L^−1^ (NH_4_)_2_ H-citrate;
  o 4.2 g L^−1^ Na_2_SO_4_;
  o 8.4 g L^−1^ (NH_4_)_2_SO_4_;
  o 1.06 g L^−1^ NH_4_Cl;
  o 31.6 g L^−1^ K_2_HPO_4_;
  o 7.4 g L^−1^ NaH_2_PO_4_·H_2_O;
- 4.5 mL of trace element solution, consisting of:
  o 0.18 g L^−1^ CoCl_2_·6 H_2_O;
  o 0.50 g L^−1^ CaCl_2_·2 H_2_O;
  o 0.18 g L^−1^ ZnSO_4_·7 H_2_O;
  o 0.10 g L^−1^ MnSO_4_·H_2_O;
  o 10.05 g L^−1^ Na_2_-EDTA·2 H_2_O;
  o 8.35 g L^−1^ FeCl_3_·6 H_2_O;
  o 0.16 g L^−1^ CuSO_4_·5 H_2_O;
- 2.2 mL of 1 mol L^−1^ MgSO_4_·7 H_2_O stock solution;
- 473.3 mL of sterile deionized H_2_O.

## Appendix B

### Appendix B.1. Additional Cultivation Results

#### Appendix B.1.1. Bacteria—*E. coli*

Complementing the online data shown in Figure 2, an offline growth curve is depicted in Figure A1. For the experiment in LB medium, a maximum specific growth rate of µ_offline_ = 1.323 h^−1^ was observed. When comparing the offline measurements with the backscattered light curve, the problem of non-linear correlation becomes apparent, which can lead to deviations in the calculation of the growth rate (µ_BSL, auto._ = 0.7561 h^−1^).

The calculation based on the OUR proved to be much more robust. Neither the size and type of the flasks, nor the process conditions, exhibited an impact on the OUR-based growth rate or its estimation. To further test the approach, a temperature variation using the TB medium was carried out (Figure A2). For the selected range (20 to 37 °C), which covers typical cultivation conditions for *E. coli* in production processes, a linear relationship between temperature and growth rate was found.

#### Appendix B.1.2. Animal Cell Cultures

As a supplement to the CHO experiments, cultivations with HEK and High Five cells were additionally performed. The results of these experiments were comparable to the above-described CHO cell experiments (Figure A3). It was shown that neither the aggregation tendency of HEK cells nor the changed shaker atmosphere for High Five cells impacted the evaluation. At µ_OUR, auto._ = 0.035 h^−1^, the algorithm-based growth rate was in very good agreement with the manually determined µ_OUR, man._ = 0.0359 h^−1^ and the µ_VCD, offline_ with 0.0350 h^−1^ (Figure A3A). The metabolic shift of lactate formation to consumption at 100 h was clearly visible in the pH signal and matched the maximum OUR and lowest O_2_ concentration. For the High Five cells, µ_OUR, auto._ = 0.0421 h^−1^, µ_OUR, man._ = 0.0436 h^−1^, and µ_VCD, offline_ = 0.0431 h^−1^ were also nearly identical.

The determined OUR-based growth rates for HEK cells (6 flasks) were µ_VCD, offline_ = 0.0315 ± 0.0026 h^−1^, µ_OUR, man._ = 0.0365 ± 0.0010 h^−1^, and µ_OUR, auto_ = 0.0355 ± 0.0005 h^−1^. At 9.863 ± 3.76 × 10^−14^ mol cell^−1^ h^−1^, the q_O2_ was the lowest of all the investigated animal cells (slightly lower than values reported by Martinez-Monge of 1.3 to 1.58 × 10^−13^ mol cell^−1^ h^−1^ [84]). The Y_X/S_ of 1.3921 ± 0.1511 × 10^6^ cells g_Glc_^−1^ was also lower than the CHO cultivation yields. The High Five cells showed the highest growth rates of all the animal cells with µ_VCD, offline_ = 0.0445 ± 0.0014 h^−1^, µ_OUR, man._ = 0.0453 ± 0.0017 h^−1^, and µ_OUR, auto_ = 0.0430 ± 0.0010 h^−1^. This was combined with the highest q_O2_ of 4.185 ± 0.024 × 10^−13^ mol cell^−1^ h^−1^ and the lowest Y_X/S_ of 1.2737 ± 0.0566 × 10^6^ cells g_Glc_^−1^. The q_O2_ lies in between the reported value for High Five cells in ExpressFive media, namely, 2.88 × 10^−13^ mol cell^−1^ h^−1^ from Rhiel et al. [74] and 9.00 × 10^−13^ mol cell^−1^ h^−1^ from Ghasemi et al. [75].

## References

[1] Arrowsmith J., Miller P. (2013). Phase II and Phase III Attrition Rates 2011–2012. Nat. Rev. Drug Discov..

[2] Paul S.M., Mytelka D.S., Dunwiddie C.T., Persinger C.C., Munos B.H., Lindborg S.R., Schacht A.L. (2010). How to Improve R & D Productivity: The Pharmaceutical Industry’s Grand Challenge. Nat. Rev. Drug Discov..

[3] US Food and Drug Administration (2009). Q8(R2)-Pharmaceutical-Development.

[4] (2015). ICH Guideline Q9 on Quality Risk Management.

[5] (2015). ICH Guideline Q10 on Pharmaceutical Quality System-Step 5.

[6] US Food and Drug Administration (2004). Guidance for Industry PAT—A Framework for Innovative Pharmaceutical Manufacturing and Quality Assurance.

[7] Katz P., Campbell C. (2012). FDA 2011 Process Validation Guidance: Process Validation Revisited. J. GXP Compliance.

[8] Borchert D., Suarez-Zuluaga D.A., Thomassen Y.E., Herwig C. (2020). Risk Assessment and Integrated Process Modeling–an Improved QbD Approach for the Development of the Bioprocess Control Strategy. AIMS Bioeng..

[9] Politis S.N., Colombo P., Colombo G., Rekkas D.M. (2017). Design of Experiments (DoE) in Pharmaceutical Development. Drug Dev. Ind. Pharm..

[10] Burdick R.K., LeBlond D.J., Pfahler L.B., Quiroz J., Sidor L., Vukovinsky K., Zhang L. (2017). Process Design: Stage 1 of the FDA Process Validation Guidance. Statistical Applications for Chemistry, Manufacturing and Controls (CMC) in the Pharmaceutical Industry.

[11] Abu-Absi S.F., Yang L., Thompson P., Jiang C., Kandula S., Schilling B., Shukla A.A. (2010). Defining Process Design Space for Monoclonal Antibody Cell Culture. Biotechnol. Bioeng..

[12] Hakemeyer C., McKnight N., John R.S., Meier S., Trexler-Schmidt M., Kelley B., Zettl F., Puskeiler R., Kleinjans A., Lim F. (2016). Process Characterization and Design Space Definition. Biologicals.

[13] Zurdo J., Arnell A., Obrezanova O., Smith N., Gómez de la Cuesta R., Gallagher T.R.A., Michael R., Stallwood Y., Ekblad C., Abrahmsén L. (2015). Early Implementation of QbD in Biopharmaceutical Development: A Practical Example. BioMed Res. Int..

[14] Zurdo J. (2013). Developability Assessment as an Early De-Risking Tool for Biopharmaceutical Development. Pharm. Bioprocess..

[15] Herwig C., Garcia-Aponte O.F., Golabgir A., Rathore A.S. (2015). Knowledge Management in the QbD Paradigm: Manufacturing of Biotech Therapeutics. Trends Biotechnol..

[16] Kumar D., Batra J., Komives C., Rathore A. (2019). QbD Based Media Development for the Production of Fab Fragments in *E. Coli*. Bioengineering.

[17] Rathore A.S., Chopda V.R., Gomes J. (2016). Knowledge Management in a Waste Based Biorefinery in the QbD Paradigm. Bioresour. Technol..

[18] Goey C.H., Bell D., Kontoravdi C. (2019). CHO Cell Cultures in Shake Flasks and Bioreactors Present Different Host Cell Protein Profiles in the Supernatant. Biochem. Eng. J..

[19] Winkler K., Socher M.L., Flickinger M.C. (2014). Shake Flask Technology. Encyclopedia of Industrial Biotechnology.

[20] Klöckner W., Büchs J. (2012). Advances in Shaking Technologies. Trends Biotechnol..

[21] Maschke R.W., Seidel S., Bley T., Eibl R., Eibl D. (2022). Determination of Culture Design Spaces in Shaken Disposable Cultivation Systems for CHO Suspension Cell Cultures. Biochem. Eng. J..

[22] Schirmer C., Maschke R.W., Pörtner R., Eibl D. (2021). An Overview of Drive Systems and Sealing Types in Stirred Bioreactors Used in Biotechnological Processes. Appl. Microbiol. Biotechnol..

[23] Xu P., Clark C., Ryder T., Sparks C., Zhou J., Wang M., Russell R., Scott C. (2017). Characterization of TAP Ambr 250 Disposable Bioreactors, as a Reliable Scale-down Model for Biologics Process Development: Characterization and Application of Ambr 250 in Clone Selection and Process Development. Biotechnol. Prog..

[24] Frey L.J., Krull R. (2020). Microbioreactors for Process Development and Cell-Based Screening Studies. Advances in Biochemical Engineering/Biotechnology.

[25] Birgen C., Degnes K.F., Markussen S., Wentzel A., Sletta H. (2021). Butanol Production from Lignocellulosic Sugars by Clostridium Beijerinckii in Microbioreactors. Biotechnol. Biofuels.

[26] Zhu Z., Hu Y., Teixeira P.G., Pereira R., Chen Y., Siewers V., Nielsen J. (2020). Multidimensional Engineering of Saccharomyces Cerevisiae for Efficient Synthesis of Medium-Chain Fatty Acids. Nat. Catal..

[27] van Dijk M., Trollmann I., Saraiva M.A.F., Brandão R.L., Olsson L., Nygård Y. (2020). Small Scale Screening of Yeast Strains Enables High-Throughput Evaluation of Performance in Lignocellulose Hydrolysates. Bioresour. Technol. Rep..

[28] Brunner M., Doppler P., Klein T., Herwig C., Fricke J. (2018). Elevated PCO_2_ Affects the Lactate Metabolic Shift in CHO Cell Culture Processes. Eng. Life Sci..

[29] Basan M., Hui S., Okano H., Zhang Z., Shen Y., Williamson J.R., Hwa T. (2015). Overflow Metabolism in *Escherichia Coli* Results from Efficient Proteome Allocation. Nature.

[30] Kleman G.L., Strohl W.R. (1994). Acetate Metabolism by *Escherichia coli* in High-Cell-Density Fermentation. Appl. Environ. Microbiol..

[31] Wolfe A.J. (2005). The Acetate Switch. Microbiol. Mol. Biol. Rev..

[32] De Mey M., De Maeseneire S., Soetaert W., Vandamme E. (2007). Minimizing Acetate Formation in *E. Coli* Fermentations. J. Ind. Microbiol. Biotechnol..

[33] Anderlei T., Zang W., Papaspyrou M., Büchs J. (2004). Online Respiration Activity Measurement (OTR, CTR, RQ) in Shake Flasks. Biochem. Eng. J..

[34] Hansen S., Hariskos I., Luchterhand B., Büchs J. (2012). Development of a Modified Respiration Activity Monitoring System for Accurate and Highly Resolved Measurement of Respiration Activity in Shake Flask Fermentations. J. Biol. Eng..

[35] Munch G., Schulte A., Mann M., Dinger R., Regestein L., Rehmann L., Büchs J. (2020). Online Measurement of CO_2_ and Total Gas Production in Parallel Anaerobic Shake Flask Cultivations. Biochem. Eng. J..

[36] Wewetzer S.J., Kunze M., Ladner T., Luchterhand B., Roth S., Rahmen N., Kloß R., Costa e Silva A., Regestein L., Büchs J. (2015). Parallel Use of Shake Flask and Microtiter Plate Online Measuring Devices (RAMOS and BioLector) Reduces the Number of Experiments in Laboratory-Scale Stirred Tank Bioreactors. J. Biol. Eng..

[37] Meier K., Klöckner W., Bonhage B., Antonov E., Regestein L., Büchs J. (2016). Correlation for the Maximum Oxygen Transfer Capacity in Shake Flasks for a Wide Range of Operating Conditions and for Different Culture Media. Biochem. Eng. J..

[38] Lapierre F.M., Schmid J., Ederer B., Ihling N., Büchs J., Huber R. (2020). Revealing Nutritional Requirements of MICP-Relevant Sporosarcina Pasteurii DSM33 for Growth Improvement in Chemically Defined and Complex Media. Sci. Rep..

[39] Brillet F., Cregut M., Durand M.J., Sweetlove C., Chenèble J.C., L’Haridon J., Thouand G. (2018). Biodegradability Assessment of Complex Chemical Mixtures Using a Carbon Balance Approach. Green Chem..

[40] Bruder S., Reifenrath M., Thomik T., Boles E., Herzog K. (2016). Parallelised Online Biomass Monitoring in Shake Flasks Enables Efficient Strain and Carbon Source Dependent Growth Characterisation of Saccharomyces Cerevisiae. Microb. Cell Factories.

[41] Sörensen M., Khakimov B., Nurjadi D., Boutin S., Yi B., Dalpke A.H., Eigenbrod T. (2020). Comparative Evaluation of the Effect of Different Growth Media on in Vitro Sensitivity to Azithromycin in Multi-Drug Resistant Pseudomonas Aeruginosa Isolated from Cystic Fibrosis Patients. Antimicrob. Resist. Infect. Control..

[42] Vasala A., Panula J., Bollók M., Illmann L., Hälsig C., Neubauer P. (2006). A New Wireless System for Decentralised Measurement of Physiological Parameters from Shake Flasks. Microb. Cell Factories.

[43] Spichiger S., Spichiger-Keller U.E., Eibl R., Eibl D. (2011). New Single-Use Sensors for Online Measurement of Glucose and Lactate: The Answer to the PAT Initiative. Single-Use Technology in Biopharmaceutical Manufacture.

[44] Ebert F.V., Reitz C., Cruz-Bournazou M.N., Neubauer P. (2018). Characterization of a Noninvasive On-Line Turbidity Sensor in Shake Flasks for Biomass Measurements. Biochem. Eng. J..

[45] Schmidt-Hager J., Ude C., Findeis M., John G.T., Scheper T., Beutel S. (2014). Noninvasive Online Biomass Detector System for Cultivation in Shake Flasks. Eng. Life Sci..

[46] Pretzner B., Maschke R.W., Haiderer C., John G.T., Herwig C., Sykacek P. (2021). Predictive Monitoring of Shake Flask Cultures with Online Estimated Growth Models. Bioengineering.

[47] Johnstone I.M., Titterington D.M. (2009). Statistical Challenges of High-Dimensional Data. Philos. Trans. R. Soc. Math. Phys. Eng. Sci..

[48] Savitzky A., Golay M.J.E. (1964). Smoothing and Differentiation of Data by Simplified Least Squares Procedures. Anal. Chem..

[49] Scipy Signal Savgol_filter—SciPy v1.8.1 Manual. https://docs.scipy.org/doc/scipy/reference/generated/scipy.signal.savgol_filter.html.

[50] Suresh S., Srivastava V., Mishra I. (2009). Techniques for Oxygen Transfer Measurement in Bioreactors: A Review. J. Chem. Technol. Biotechnol..

[51] Bauer I., Dreher T., Eibl D., Glöckler R., Husemann U., John G.T., Kaiser S.C., Kampeis P., Kauling J., Kleebank S. (2020). Recommendations for Process Engineering Characterisation of Single-Use Bioreactors and Mixing Systems by Using Experimental Methods.

[52] Seidel S., Maschke R.W., Werner S., Jossen V., Eibl D. (2021). Oxygen Mass Transfer in Biopharmaceutical Processes: Numerical and Experimental Approaches. Chem. Ing. Tech..

[53] Werner S., Olownia J., Egger D., Eibl D. (2013). An Approach for Scale-Up of Geometrically Dissimilar Orbitally Shaken Single-Use Bioreactors. Chem. Ing. Tech..

[54] Andersen K.B., von Meyenburg K. (1980). Are Growth Rates of *Escherichia coli* in Batch Cultures Limited by Respiration?. J. Bacteriol..

[55] Lin H.Y., Mathiszik B., Xu B., Enfors S.-O., Neubauer P. (2001). Determination of the Maximum Specific Uptake Capacities for Glucose and Oxygen in Glucose-Limited Fed-Batch Cultivations of *Escherichia coli*. Biotechnol. Bioeng..

[56] van der Aar P.C., van Verseveld H.W., Stouthamer A.H. (1990). Stimulated Glycolytic Flux Increases the Oxygen Uptake Rate and Aerobic Ethanol Production, during Oxido-Reductive Growth of Saccharomyces Cerevisiae. J. Biotechnol..

[57] Pépin M.-F., Archambault J., Chavarie C., Cormier F. (1995). Growth Kinetics of *Vitis Vinifera* Cell Suspension Cultures: I. Shake Flask Cultures. Biotechnol. Bioeng..

[58] Pfizenmaier J., Matuszczyk J.-C., Takors R. (2015). Changes in Intracellular ATP-Content of CHO Cells as Response to Hyperosmolality. Biotechnol. Prog..

[59] Aehle M., Kuprijanov A., Schaepe S., Simutis R., Lübbert A. (2011). Simplified Off-Gas Analyses in Animal Cell Cultures for Process Monitoring and Control Purposes. Biotechnol. Lett..

[60] Biener R., Steinkämper A., Hofmann J. (2010). Calorimetric Control for High Cell Density Cultivation of a Recombinant *Escherichia coli* Strain. J. Biotechnol..

[61] Xu B., Jahic M., Enfors S.-O. (1999). Modeling of Overflow Metabolism in Batch and Fed-Batch Cultures of *Escherichia coli*. Biotechnol. Prog..

[62] Stahl G., Salem S.N.B., Chen L., Zhao B., Farabaugh P.J. (2004). Translational Accuracy during Exponential, Postdiauxic, and Stationary Growth Phases in *Saccharomyces cerevisiae*. Eukaryot. Cell.

[63] du Preez J.C., Maré J.E., Albertyn J., Kilian S.G. (2001). Transcriptional Repression of ADH2-Regulated β-Xylanase Production by Ethanol in Recombinant Strains of *Saccharomyces cerevisiae*. FEMS Yeast Res..

[64] Paalme T., Elken R., Vilu R., Korhola M. (1997). Growth Efficiency of *Saccharomyces cerevisiae* on Glucose/Ethanol Media with a Smooth Change in the Dilution Rate (A-Stat). Enzym. Microb. Technol..

[65] Lei F., Olsson L., Jørgensen S.B. (2004). Dynamic Effects Related to Steady-State Multiplicity in Continuous *Saccharomyces Cerevisiae* Cultivations: Steady-State Multiplicity. Biotechnol. Bioeng..

[66] Granata T., Follonier C., Burkhardt C., Rattenbacher B. (2021). Methods for Oxygenation of Continuous Cultures of Brewer’s Yeast, Saccharomyces Cerevisiae. Fermentation.

[67] Cuperus F.S. (2007). Influence of UV-B Radiation on Quality Determination Compounds in Must and Wine and Suspension Cell Cultures of Vitis vinifera.

[68] Hirasuna T.J., Shuler M.L., Lackney V.K., Spanswick R.M. (1991). Enhanced Anthocyanin Production in Grape Cell Cultures. Plant Sci..

[69] Möller J., Bhat K., Guhl L., Pörtner R., Jandt U., Zeng A. (2021). Regulation of Pyruvate Dehydrogenase Complex Related to Lactate Switch in CHO Cells. Eng. Life Sci..

[70] Arndt L., Wiegmann V., Kuchemüller K.B., Baganz F., Pörtner R., Möller J. (2021). Model-based Workflow for Scale-up of Process Strategies Developed in Miniaturized Bioreactor Systems. Biotechnol. Prog..

[71] Möller J., Kuchemüller K.B., Steinmetz T., Koopmann K.S., Pörtner R. (2019). Model-Assisted Design of Experiments as a Concept for Knowledge-Based Bioprocess Development. Bioprocess Biosyst. Eng..

[72] Maschke R.W., Eibl D. (2019). Process Transfer of CHO Cultivations Using the Minifors 2 as an Example.

[73] Müller J., Ott V., Eibl D., Eibl R. (2022). Seed Train Intensification Using an Ultra-High Cell Density Cell Banking Process. Processes.

[74] Rhiel M., Mitchell-Logean C.M., Murhammer D.W. (1997). Comparison of *Trichoplusia ni* BTI-Tn-5B1-4 (High Five^TM^) and *Spodoptera frugiperda Sf*-9 Insect Cell Line Metabolism in Suspension Cultures. Biotechnol. Bioeng..

[75] Ghasemi A., Bozorg A., Rahmati F., Mirhassani R., Hosseininasab S. (2019). Comprehensive Study on Wave Bioreactor System to Scale up the Cultivation of and Recombinant Protein Expression in Baculovirus-Infected Insect Cells. Biochem. Eng. J..

[76] Bögli N.C., Ries C., Adams T., Greller G., Eibl D., Eibl R. (2015). Large-Scale, Insect-Cell–Based Vaccine Development. BioProInt.

[77] Castillo Salvador A.E., Fuge G., Jandt U., Zeng A.-P. (2015). Growth Kinetics and Validation of Near-Physiologically Synchronized HEK293S Cultures. Eng. Life Sci..

[78] Martínez-Monge I., Comas P., Triquell J., Lecina M., Casablancas A., Cairó J.J. (2018). A New Strategy for Fed-Batch Process Control of HEK293 Cell Cultures Based on Alkali Buffer Addition Monitoring: Comparison with O.U.R. Dynamic Method. Appl. Microbiol. Biotechnol..

[79] Fontova A., Lecina M., López-Repullo J., Martínez-Monge I., Comas P., Bragós R., Cairó J.J. (2018). A Simplified Implementation of the Stationary Liquid Mass Balance Method for On-Line OUR Monitoring in Animal Cell Cultures: Simplified OUR on-Line Monitoring Based on Stationary Liquid Mass Balance. J. Chem. Technol. Biotechnol..

[80] Sohoni S.V., Bapat P.M., Lantz A.E. (2012). Robust, Small-Scale Cultivation Platform for Streptomyces Coelicolor. Microb. Cell Factories.

[81] Anderlei T., Keebler M.V., Cairó J.J., Lecina M., Pörtner R. (2020). HEK293 Cell-Based Bioprocess Development at Bench Scale by Means of Online Monitoring in Shake Flasks (RAMOS and SFR). Animal Cell Biotechnology.

[82] Lennox E.S. (1955). Transduction of Linked Genetic Characters of the Host by Bacteriophage P1. Virology.

[83] (2015). Terrific Broth (TB) Medium. *Cold Spring Harb. Protoc.*. http://cshprotocols.cshlp.org/content/2015/9/pdb.rec085894.

[84] (2006). YPD. *Cold Spring Harbor Protocols.*. http://cshprotocols.cshlp.org/content/2006/1/pdb.rec8194.

[85] Murashige T., Skoog F. (1962). A Revised Medium for Rapid Growth and Bio Assays with Tobacco Tissue Cultures. Physiol. Plant..

[86] Zhao L., Fan L., Zhang X., Zhu M., Tan W. (2007). The Role of Microenvironment in Aggregation of the 293-Human Embryonic Kidney Cells. Korean J. Chem. Eng..

